# Comparison of the Usefulness of Optical Coherence Tomography Angiography and Fluorescein Angiography in the Diagnosis of Diabetic Macular Edema

**DOI:** 10.3390/diagnostics15151873

**Published:** 2025-07-25

**Authors:** Alfred Niewiem, Krzysztof Broniarek, Katarzyna Michalska-Małecka

**Affiliations:** 1 Zagłębiów Clinical Hospital Czeladź, Szpitalna 40, 41-250 Czeladź, Poland; niewiem.alfred@gmail.com; 2 Department of Ophthalmology, Medical University of Gdańsk, Mariana Smoluchowskiego 17, 80-001 Gdańsk, Poland; k.broniarek@gumed.edu.pl

**Keywords:** diabetic retinopathy, macular edema, DME, fluorescein angiography, coherence optical tomography angiography

## Abstract

**Background/Objectives:** Diabetic macular edema (DME) is the primary cause of vision loss in people with diabetes, and if untreated, it can result in irreversible macular damage. Both fluorescein angiography (FA), the gold standard, and optical coherence tomography angiography (OCTA) are used for evaluation of this disease. The objective of this study was to compare the diagnostic value of both. **Methods:** We conducted a comparative analysis of 98 patients aged 18–80 years with significant DME and best-corrected visual acuity ≥0.1 according to the Snellen chart. Participants underwent glycated hemoglobin blood test (HbA1c) and ophthalmological examinations, including OCTA and FA. OCTA 3 × 3 mm scans of superficial (SCP) and deep capillary plexus (DCP) along with FA scans were exported to the Gimp computer program. Size of the foveal avascular zone (FAZ), the number of visible microaneurysms (MAs), and ETDRS report number 11 classification of the images were assessed. **Results:** FAZ size differed significantly in superficial plexus (0.41 mm^2^), deep plexus (0.43 mm^2^) OCTA, and FA (0.38 mm^2^) (*p* < 0.001). FAZ size in DCP OCTA closely correlated with that of FA (τ = 0.79, *p* < 0.001). The total number of MAs visualized in the OCTA was significantly lower than in FA (*p* < 0.001). ETDRS classification of scans revealed that the level of consistency between the examinations was moderate to very strong. **Conclusions:** OCTA may be useful in evaluating macular ischemia. It is less sensitive in detecting MAs in DME eyes. FAZ has sharper boundaries and is larger when measured in OCTA. Poor glycemic control results in higher incidence of MAs in macula.

## 1. Introduction

Diabetes mellitus is a chronic condition characterized by elevated blood glucose levels and can lead to severe complications affecting multiple organs. It is estimated that approximately 100–120 million people worldwide have diabetic retinopathy, and around 20–30 million suffer from proliferative diabetic retinopathy or DME [1,2]. It is projected that by 2045, the global prevalence of diabetes will reach 629 million, compared to 108 million in 1980—a nearly sixfold increase. Notably, this surge is expected to be most pronounced in low- and middle-income countries [3]. A large proportion of individuals with diabetes are of working age, and in this demographic group, DME is the most frequent cause of blindness. Consequently, the social and economic implications of this disease are significant.

Frequent ocular complications of diabetes include diabetic retinopathy (DR), maculopathy, iridopathy, and unstable refraction. Maculopathy is subdivided into diabetic maculopathy, primarily DME, and ischemic maculopathy. Characteristic features of diabetic retinopathy include microaneurysms (MAs), retinal hemorrhages, exudates, focal/diffuse maculopathy, cotton wool spots, venous changes, intraretinal vascular abnormalities (IRMA), arterial alterations, and neovascularization at the disc (NVD) or elsewhere (NVE) [4]. While microvascular complications are hallmark signs of diabetic retinopathy, neuroretinal dysfunction often precedes these changes. The new term, functional diabetic retinopathy, emphasizes the importance of these prestructural changes. It is worth mentioning that changes like ganglion cell loss or inner retinal thinning may contribute to visual field loss, even before diabetic retinopathy is visible. In later stages of DR, DME can mask these changes, hindering accurate assessment of disease severity [5].

DME is the principal cause of decreased visual acuity in patients with diabetes. If left untreated, DME can lead to irreversible morphological and functional changes in the macula. The primary risk factors for developing DME include (1) the duration of diabetes (ranging from 0% in patients with type 1 diabetes of less than 5 years to 28–29% in patients with diabetes, both type 1 and type 2, of more than 20 years’ duration); (2) poor glycemic control—at HbA1c levels exceeding 10%, there is a rapid progression of systemic complications, including ocular manifestations; (3) uncontrolled arterial hypertension; and (4) cigarette smoking [6,7].

The Early Treatment Diabetic Retinopathy Study (ETDRS) defines clinically significant macular edema as (1) retinal thickening within 500 µm of the center of the fovea; (2) hard exudates located within 500 µm of the center accompanied by adjacent retinal thickening, which may extend beyond 500 µm; or (3) retinal thickening that involves an area of at least one disc diameter (DD) if any portion lies within 1 DD of the foveal center.

Fluorescein angiography (FA) is the gold standard for diagnosing DME. This invasive procedure employs the contrast agent fluorescein, typically administered intravenously [8]. Fluorescein is well tolerated by most patients. Adverse reactions to intravenous administration are classified as mild—the most frequent, moderate, severe, or even fatal [9]. FA facilitates real-time imaging of the posterior segment structures, allowing for the evaluation of blood flow in the retinal and choroidal vasculature. FA can detect all the aforementioned markers of diabetic retinopathy. Vascular changes such as MAs are often more conspicuous on angiographic images compared to ophthalmoscopic evaluations; however, once these lesions become occluded, they are no longer visible in FA. In images of cystoid macular edema, a characteristic petaloid pattern of hyperfluorescence is observed. In cases of ischemic maculopathy, which may not present overt signs on ophthalmoscopy, FA can reveal an enlargement of the foveal avascular zone (FAZ) [10]. Conventional angiography covers 30–50° of the retina in a single image. Recent advancements in fundus camera technology, such as wide-field angiography, allow visualization of 30–200° of the retina, while ultra-wide-field angiography extends the field of view to more than 200° [11].

The indications and role of FA have evolved due to the increasingly widespread availability of optical coherence tomography (OCT) along with OCTA function. Data obtained from both modalities may be indispensable in treatment planning, including laser therapy, intravitreal injections of corticosteroids and anti-VEGF agents, and even pars plana vitrectomy.

OCTA is a modern, noninvasive imaging technique for visualizing the retinal and choroidal vasculature. Since it does not require a contrast agent, there is no risk of severe adverse reactions. OCTA enables the assessment of the retinal vasculature in individual layers, permitting independent evaluation of both the superficial and deep vascular networks. Furthermore, integrated software allows for the quantitative analysis of vascular density in specific regions of the macula [12]. Although the evaluation of the choriocapillaris remains challenging—as in FA—OCTA is useful in analyzing this vascular layer [13]. Limitations of OCTA include its relatively small scanning area and an increased susceptibility to artifacts.

The main aim of this study was to compare the clinical usefulness of OCTA scans and fluorescein angiography in DME assessment.

## 2. Materials and Methods

### 2.1. Ethics

Approval for the study was obtained from the Bioethics Committee at the Silesian Medical University in Katowice (Resolution No. KNW/0022/KB1/128/17 dated 19 December 2017).

### 2.2. Study Design

A total of 106 patients treated at the Retina Clinic of the Department of Ophthalmology, University Clinical Center named after Prof. K. Gibiński of the Silesian Medical University in Katowice, were enrolled in the prospective study. Patients were examined according to the following scheme: (a) patient subjective assessment and medical interview, (b) autorefractometry, (c) best corrected visual acuity (BCVA) assessment, (d) intraocular pressure measurement, (e) examination of the anterior segment of the eye with a slit lamp, (f) fundus examination, (g) OCT and macular OCTA, (h) fluorescein angiography, and (i) glycated hemoglobin evaluation.

Patients provided their written informed consent to participate in the study. Each was informed about the nature of the examinations performed and the potential adverse effects. Additionally, patients received written information regarding the assumptions and course of the study.

### 2.3. Participants

The following inclusion/exclusion criteria were used: inclusion criteria: patients with significant diabetic macular edema according to the ETDRS criteria, age 18–80 years, BCVA ≥ 0.1, and exclusion criteria: other causes of macular edema, proliferatvice diabetic retinopathy, history of retinal arterial occlusion, history of retinal vein thrombosis, active or past uveitis, macular dystrophies, high myopia, history of pars plana vitrectomy, history of retinal laser therapy in the last 3 months, newly diagnosed or previously treated glaucoma, presence of epiretinal membrane, vitreoretinal traction, previous intravitreal anti-VEGF/corticosteroid therapy, advanced cataract—LOCS III scale, allergy to fluorescein, bronchial asthma, kidney failure, pregnancy, or lactation.

In 6 patients, numerous artifacts related to eye movement were observed during the analysis of the acquired OCTA images of the macula, and in 2 patients the scan quality was too low, which prevented further evaluation. Consequently, these patients were excluded from comparative analysis. Ultimately, 98 patients were included in the final statistical analysis.

### 2.4. Data Acquisition

FA was performed using the HRA Spectralis device (Heidelberg Engineering, Heidelberg, Germany), software version 6.5.2.0. The patient’s refractometric values and demographic data were entered into the system prior to the examination. The examination commenced with the eye selected for clinical analysis. At the researcher’s command, a nurse administered 5 mL of a 10% fluorescein solution via an intravenous injection in the antecubital fossa. Immediately thereafter, angiogram acquisition was initiated, covering the choroidal phase through to the late phase. Following the examination, the patient was observed for one hour for any potential adverse reactions. During the early-phase angiograms, quantitative parameters—such as the number of microaneurysms (MAs) and the area of the foveal avascular zone (FAZ)—were manually determined using the device software. In addition, the angiograms were evaluated according to the classification criteria outlined in ETDRS report number 11 for the following parameters: FAZ size, FAZ outline, and capillary loss.

Optical coherence tomography (OCT) and OCTA were performed using the AngioVue device (Optovue, Fremont, CA, USA), software version 2018.1.1.60. Prior to initiation, the patient’s refractometric values were input into the system to enhance scan quality. Qualitative assessments included the evaluation of disorganization of the retinal inner layers, the presence of subretinal fluid, epiretinal membranes, or vitreoretinal traction. Quantitatively, the central retinal thickness (CRT) under foveal fixation and the maximum retinal thickness (max RT) were objectively measured. In the OCTA examination, automatic processing provided quantitative data such as vascular density (VD) in both the superficial (SCP) and deep (DCP) capillary plexuses. Conversely, parameters including the number of MAs and the FAZ area in the SCP and DCP were manually counted. Qualitative evaluation of OCTA scans also addressed the presence of interconnections (shunts) between the SCP and DCP. Furthermore, in accordance with the ETDRS report number 11 classification, OCTA scans—in both the SCP and DCP—were assessed for FAZ size, FAZ outline, and capillary loss. All OCT and OCTA scans were evaluated for image quality based on the signal strength index (SSI) on a scale from 1 to 100 as well as for artifacts related to ocular movements. In cases of image degradation or SSI values below 37, the examination was repeated, and if high-quality scans could not be obtained, the examined eye was excluded from further analysis. Automatic segmentation of macular layers was applied in all scans.

The OCT examination employed the following scanning protocols:

- 3D retina (retina module): Each examination comprised 141 macular cross-sections at a resolution of 640 × 385 pixels, representing a macular volume of 2 × 7 × 7 mm.
- Retina map (retina module): This protocol is based on a grid pattern covering an outer area of 6 × 6 mm with 13 vertical and 13 horizontal scans at 0.5 mm intervals as well as an inner area of 4 × 4 mm with 8 vertical and 8 horizontal scans at 0.5 mm intervals.
- AngioVue Retina 3.0 mm (AngioVue module): This examination permits analysis of blood flow in all macular vascular layers with simultaneous visualization of en face images and OCT scans. Each OCTA exam comprises 304 × 304 A-scans with two consecutive B-scans acquired at each fixation point before progressing to subsequent areas. The registered B-scans are then compared to calculate decorrelation and eliminate artifacts resulting from ocular motion. The scanning area measures 3 × 3 mm with the fixation point centered in the fovea.

### 2.5. Preparation of Images for Evaluation

AngioVue Retina 3.0 mm scans of the superficial capillary plexus (SCP) and deep capillary plexus (DCP), as well as 20° angiograms, were exported to GIMP V.2.10.24 (gimp.org). A new layer was then created consisting of two circles—an inner circle with a radius corresponding to 500 µm and an outer circle with a radius of 1500 µm. This layer represents the inner ring of the ETDRS grid, divided into 5 smaller segments: central, superior, inferior, nasal, and temporal. The created layer was manually overlaid onto the en face OCTA scans and the FA images, which were magnified and cropped to spatially correspond with the 3 × 3 mm OCTA area. Corresponding regions from both examinations were analyzed for the number of microaneurysms (MAs) and classified according to the parameters in ETDRS report number 11. Arterial abnormalities were not evaluated because their identification in OCTA images was often ambiguous. The FAZ outline was assessed as follows: Grade 0 = normal outline; Grade 1 = contour not completely round or oval, with minor irregularities that are not necessarily pathological; Grade 2 = visible FAZ defects, <180°; Grade 3 = obviously damaged FAZ, >180°; Grade 4 = completely disrupted FAZ outline; Grade 8 = not assessable. Capillary loss was evaluated within the central field as well as in the four remaining fields (superior, inferior, temporal, and nasal) using the following scale: Grade 0 = no loss; Grade 1 = doubtful loss; Grade 2 = mild loss (up to 25% loss within subfields); Grade 3 = moderate loss (>25% and up to 50% loss within subfields); Grade 4 = severe loss (>50% loss within subfields); Grade 8 = not assessable. MA counting was also performed for all considered subfields. MAs were defined as distinctly round, saccular, or spindle-shaped hyperfluorescent points visible in the early phases of FA and OCTA as localized vascular dilations connected to the retinal capillary network.

The FAZ was manually evaluated using the device’s built-in software for both the fundus camera (FA) and the OCTA system. Once the foveal avascular zone outline was delineated, the software provided a measurement in square millimeters, which was used for further analysis.

### 2.6. HbA1c Measurement

HbA1c concentration was determined using high-performance liquid chromatography (HPLC) on full venous blood collected in ethylenediaminetetraacetic acid (EDTA). The measurement result was reported in accordance with the National Glycohemoglobin Standardization Program standard (NGSP), expressed as a percentage with reference values of 4.2–6.3%.

### 2.7. Statistical Analysis

Statistical computations were performed using R v.4.1.1 and RStudio IDE v.1.4.1717 (R Core Team, 2021 [14]). A significance level of α = 0.05 was adopted for the statistical tests in this study. For interval-scale variables, grouped descriptive statistics were employed to describe the study sample and to draw basic conclusions and generalizations regarding the cohorts. For this purpose, the built-in function described by () from the {psych} package was used [15]. Additionally, normality was assessed using the Shapiro–Wilk test, considering the test statistic W and the corresponding *p*-value (via the shapiro.test () function from the {stats} package) [14].

## 3. Results

### 3.1. Descriptive Statistics of the Study Group

The analyzed dataset presented in Table 1 consists of 98 observations. The Shapiro–Wilk test indicated that the distributions of OCTA VD 1 and OCTA VD 2 were normal. The remaining variables did not follow a normal distribution, including age, BCVA, CRT, Max RT, HbA1c, OCTA FAZ 1, OCTA FAZ 2, OCTA MAs 1, OCTA MAs 2, FA FAZ, FA MAs, duration of diabetes (in years), and duration of vision impairment (in months).

#### 3.1.1. Age

In the study, there were 47 female participants with an average age of 64.26 years and a standard deviation of 6.37 years. The median age for the women was 65 years, with ages ranging from 49 to 76 years, and the distribution showed a skewness of −0.30 and a kurtosis of −0.69. Among the 51 male participants, the mean age was 63.71 years with a standard deviation of 7.79 years. Their median age was also 65 years, and their ages varied from 36 to 75 years. The age distribution for the men exhibited a skewness of −1.54 and a kurtosis of 3.03. The Shapiro–Wilk test revealed that the distribution of the age variable deviated from a normal distribution.

#### 3.1.2. Distribution of Patient Age Groups by Gender

In the group of women, 22 individuals (46.8%) were under 65 years of age and 25 (53.2%) were 65 years or older, totaling 47 women. The chi-square test for women (df = 1) resulted in χ^2^ = 0.00, *p* = 0.987, with a Cramér’s V (φc) of 0.02. Among men, 25 (49.0%) were under 65 years of age and 26 (51.0%) were 65 years or older, making up a total of 51 men. Overall, the study included 90 individuals, with 47 (48.0%) under 65 and 51 (52.0%) who were 65 years or older. No association was observed between gender and age group; the frequencies of gender distributions across age groups did not differ significantly.

#### 3.1.3. Duration of Diabetes

In the study sample of 98 individuals, the mean diabetes duration was as follows: for women (*n* = 47)—14.34 years (SD = 9.14, median = 15, range: 1–33 years, skewness = 0.17, kurtosis = −0.86) and for men (*n* = 51)—16.01 years (SD = 8.29, median = 15, range: 0.7–35 years, skewness = 0.09, kurtosis = −0.62). The Shapiro–Wilk test indicated that the distribution of the diabetes duration variable was different from the normal distribution.

#### 3.1.4. Glycosylated Hemoglobin Concentration

Distribution of glycosylated hemoglobin concentration [%] by sex (*N* = 98) was for women (*n* = 47): mean = 7.73, SD = 1.53, median = 7.5, range = 5.4–11.4, skewness = 0.57, kurtosis = −0.22) and for men (*n* = 51): mean = 7.87, SD = 1.44, median = 7.7, range = 5.2–11.2, skewness = 0.47, kurtosis = −0.09. The Shapiro–Wilk test indicated that the distribution of glycosylated hemoglobin concentration was different from a normal distribution.

#### 3.1.5. Mean Best Corrected Visual Acuity

Distribution of corrected visual acuity by age group (*N* = 98) presented as follows: <65 years: *n* = 47, M = 0.57, SD = 0.15, median = 0.6, range: 0.2–0.8, skewness = −0.27, kurtosis = −0.4 and 65 years or older: *n* = 51, M = 0.52, SD = 0.13, median = 0.5, range: 0.2–0.8, skewness = −0.50, kurtosis = 0.39. The Shapiro–Wilk test indicated that the distribution of the corrected visual acuity variable was different from a normal distribution.

#### 3.1.6. Central Retinal Thickness (CRT), Maximum Retinal Thickness (Max RT)

The distribution of the mean measures of the ratio scale variables in the case of CRT [µm] for women (*n* = 47) was M = 409.49, SD = 108.02, median = 392, range = 220–790, skewness = 1.29, kurtosis = 2.73 and for men (*n* = 51) was M = 443.71, SD = 108.84, median = 410, range = 294–786, skewness = 1.28, kurtosis = 1.28.

The distribution of the mean measures of the ratio scale variables, in case of Max RT [µm], for women (*n* = 47) was M = 471.62, SD = 97.26, median = 448, range = 361–790, skewness = 1.16, kurtosis = 1.22 and for men (*n* = 51) was M = 501.65, SD = 105.27, median = 488, range = 332–786, skewness = 0.89, kurtosis = 0.29. The Shapiro–Wilk test indicated that the distributions of the CRT and Max RT variables were different from a normal distribution.

#### 3.1.7. Smoking Status

In the study sample of 98 participants, there were 47 women (48%) and 51 men (52%). Among non-smokers, 33 women (48.5%) and 35 men (51.5%) were observed, totaling 68 individuals. For the smoker group, there were 14 women (46.7%) and 16 men (53.3%), making up 30 individuals. An independence test was conducted with 1 degree of freedom, yielding a chi-square value of 0.00, a *p*-value of 1.000, and a phi coefficient of 0.02, indicating no significant association between sex and smoking status.

#### 3.1.8. Prevalence of Arterial Hypertension

In the study of 98 participants, 47 individuals (48%) were younger than 65 years, while 51 (52%) were 65 years or older. Among the participants without hypertension, all 21 individuals (100%) were younger than 65 years, with none in the older group. In contrast, among those with hypertension, 26 individuals (33.8%) were under 65 years, and 51 (66.2%) were 65 or older, totaling 77 with hypertension. An independent chi-square test (df = 1) revealed a chi-square value of 26.41, with a *p*-value of <0.001 and a phi coefficient of 0.54, indicating a statistically significant association between age group and hypertension status. A statistically significant association of moderate strength was demonstrated between arterial hypertension and the age group. It was observed that all cases of hypertension were found in the “65 years and older” group.

### 3.2. Implementation of the Main Objectives of the Conducted Research

#### 3.2.1. Comparison of the Number of Microaneurysms Assessed Using Different Methods

The distribution of the mean measures for the number of microaneurysms counted using different methods is presented as follows (*N* = 98):

For OCTA MAs 1, the mean is 0.84 with a standard deviation of 1.60. The median is 0.00 with an interquartile range of 1.00, and the values range from 0.00 to 14.00. The distribution is highly skewed (skewness = 5.73) and shows very high kurtosis (43.56), with a Shapiro–Wilk statistic of 0.46 (*p* < 0.001).

For OCTA MAs 2, the mean is 2.41 with a standard deviation of 2.27. The median is 2.00, and the interquartile range is 4.00, with a range from 0.00 to 8.00. This distribution has a moderate skewness (0.54) and a slight platykurtic tendency (kurtosis = −0.91), with a Shapiro–Wilk statistic of 0.88 (*p* < 0.001).

When combining OCTA MAs 1 and OCTA MAs 2, the mean measure increases to 3.24 with a standard deviation of 3.32. The median is 3.00, and the interquartile range is 9.00, with values spanning from 0.00 to 19.00. The distribution exhibits moderate skewness (1.39) and a kurtosis of 3.40, with a Shapiro–Wilk test value of 0.90 (*p* < 0.001).

For FA MAs, the mean is 5.47 with a standard deviation of 4.93. The median is 5.00, and the interquartile range is 6.00, with a minimum value of 0.00 and a maximum of 17.00. This distribution shows slight skewness (0.47) and moderate kurtosis (−1.01), with a Shapiro–Wilk statistic of 0.85 (*p* < 0.001). The Shapiro–Wilk test revealed that the distribution deviated from normality; therefore, a nonparametric test was employed during the analysis of differences. The significance of the differences between individual groups was assessed using a nonparametric test for paired observations.

#### 3.2.2. The Significance of Differences Between the OCTA MAs 1 and FA MAs Variables

A nonparametric Wilcoxon test for paired samples indicated that the difference between the median count of FA MAs (Mdn = 5.00; IQR = 9.00) and the median count of OCTA MAs 1 (Mdn = 0.00; IQR = 1.00) was statistically significant, VWilcoxon = 2500, *p* < 0.001, with a very high correlation between the variables (ρ = 0.99).

The significance of differences between the variables OCTA MAs 2 and FA MAs was also examined. A nonparametric Wilcoxon test for paired samples showed that the difference between the median count of FA MAs (Mdn = 5.00; IQR = 9.00) and the median count of OCTA MAs 2 (Mdn = 2.00; IQR = 4.00) was statistically significant, VWilcoxon = 2600, *p* < 0.001, with a perfect correlation between the variables (ρ = 1.00).

#### 3.2.3. The Significance of Differences Between the OCTA MAs 1 + OCTA MAs 2 a FA MAs Variables

A non-parametric Wilcoxon signed-rank test revealed a statistically significant difference between the median number of microaneurysms (MAs) identified using fluorescein angiography (FA MAs; Mdn = 5.00, IQR = 9.00) and the median combined number of MAs detected in OCTA1 and OCTA2 (Mdn = 3.00, IQR = 6.00), *V_Wilcoxon_* = 2400, *p* < 0.001, indicating a very strong association between the variables, $\hat{r_{biserial}^{rank}}$ = 0.94.

#### 3.2.4. Assessment of the Significance of Differences in FAZ Area Between the Superficial and Deep Plexus on OCTA

A non-parametric Wilcoxon signed-rank test demonstrated a statistically significant difference between the median FAZ area in the superficial plexus (OCTA1: Mdn = 0.41 mm^2^; IQR = 0.09) and the deep plexus (OCTA2: Mdn = 0.43 mm^2^; IQR = 0.14), *V_Wilcoxon_* = 12.50, *p* < 0.001, indicating a very strong association between the variables, $\hat{r_{biserial}^{rank}}$= −0.99.

#### 3.2.5. Assessment of the Significance of Differences in FAZ Area Between the Superficial Plexus on OCTA and Fluorescein Angiography

A non-parametric Wilcoxon signed-rank test showed a statistically significant difference between the median FAZ area measured by superficial OCTA (OCTA1: Mdn = 0.41 mm^2^; IQR = 0.40) and that measured by fluorescein angiography (FA: Mdn = 0.38 mm^2^; IQR = 0.40), *V_Wilcoxon_* = 4400, *p* < 0.001, indicating a very strong association between the variables, $\hat{r_{biserial}^{rank}}$ = 0.91.

#### 3.2.6. Assessment of the Significance of Differences in FAZ Area Between the Deep Plexus on OCTA and Fluorescein Angiography

A non-parametric Wilcoxon signed-rank test revealed a statistically significant difference between the median FAZ area in the deep OCTA layer (OCTA2: Mdn = 0.43 mm^2^; IQR = 0.39) and the median FAZ area measured by fluorescein angiography (FA: Mdn = 0.38 mm^2^; IQR = 0.40), *V_Wilcoxon_* = 4700, *p* < 0.001, indicating a perfect association between the variables, $\hat{r_{biserial}^{rank}}$= 1.00.

It was demonstrated that the absolute value of the rank-based point-biserial correlation was higher for the comparison between FAZ OCTA2 and FAZ FA (1.0) than for the comparison between FAZ OCTA1 and FAZ FA (0.91).

#### 3.2.7. Assessment of the Correlation Between the Degree of Macular Ischemia in Fluorescein Angiography and OCTA According to the ETDRS Report No. 11 Classification in the “Size of FAZ” Category

Kendall’s tau correlation was used as the method for assessing relationships between variables measured on an ordinal scale. Kendall’s tau correlation coefficient for the variable pair OCTA size of FAZ 1 vs. OCTA size of FAZ 2 was τ = 0.89, *p* < 0.001. For the pair OCTA size of FAZ 1 vs. FA size of FAZ, the coefficient was τ = 0.88, *p* < 0.001. For the pair OCTA size of FAZ 2 vs. FA size of FAZ, the coefficient was τ = 0.79, *p* < 0.001.

The data analyzed demonstrated a strong, statistically significant positive association between all variables. An increase in one variable was accompanied by a closely corresponding increase in the other variable (and vice versa).

#### 3.2.8. Assessment of the Correlation in the Degree of Macular Ischemia Between Fluorescein Angiography and OCTA According to the ETDRS Report No. 11 Classification in the “Outline of FAZ” Category

Kendall’s tau correlation was used to assess relationships between variables measured on an ordinal scale. Kendall’s tau correlation coefficient for the variable pair OCTA Outline of FAZ 1 vs. OCTA Outline of FAZ 2 was τ = 1.00, *p* < 0.001.

For the pair OCTA Outline of FAZ 1 vs. FA Outline of FAZ, the coefficient was τ = 0.91, *p* < 0.001. For the pair OCTA Outline of FAZ 2 vs. FA Outline of FAZ, the coefficient was τ = 0.91, *p* < 0.001.

A perfect or very strong statistically significant association was observed between all variables. An increase in one variable corresponded to an increase in the other variable (and vice versa), with values that were either nearly identical or very close. The relationship between the variables followed a pattern resembling a straight line.

#### 3.2.9. Assessment of the Correlation in the Degree of Macular Ischemia Between Fluorescein Angiography and OCTA According to the ETDRS Report No. 11 Classification in the “Capillary Loss” Category

Kendall’s tau correlation was used to assess relationships between variables measured on an ordinal scale. 

Kendall’s tau correlation coefficient for the variable pair OCTA Capillary Loss 1 vs. OCTA Capillary Loss 2 was τ = 0.74, *p* < 0.001. For the pair OCTA Capillary Loss 1 vs. FA Capillary Loss, the coefficient was τ = 0.93, *p* < 0.001. For the pair OCTA Capillary Loss 2 vs. FA Capillary Loss, the coefficient was τ = 0.68, *p* < 0.001.

Based on the above-mentioned data, a statistically significant association was observed between all variables, ranging from moderate (0.68) to very strong (0.93). An increase in one variable corresponded to an increase in the other variable (and vice versa).

#### 3.2.10. Assessment of Agreement Between Fluorescein Angiography and OCTA in Evaluating the Degree of Macular Ischemia According to the ETDRS Report No. 11 Classification

To assess the agreement between fluorescein angiography (FA) and OCTA in evaluating the degree of macular ischemia based on the ETDRS Report No. 11 classification, Cohen’s kappa coefficient was used (N = 98). For OCTA1 vs. FA, Cohen’s kappa for Outline of FAZ was 0.86 with a 95% confidence interval of [0.76, 0.95]. Cohen’s kappa for Size of FAZ was 0.82 with a 95% confidence interval of [0.72, 0.93]. Cohen’s kappa for Capillarity Loss of FAZ was 0.92 with a 95% confidence interval of [0.84, 1.00].

For OCTA2 vs. FA, Cohen’s kappa for Outline of FAZ was 0.86 with a 95% confidence interval of [0.76, 0.95]. Cohen’s kappa for Size of FAZ was 0.64 with a 95% confidence interval of [0.54, 0.81]. Cohen’s kappa for Capillarity Loss of FAZ was 0.61 with a 95% confidence interval of [0.44, 0.78].

The level of agreement for the OCTA1 vs. FA pair was found to range from strong (reliable data share of 64–81%) to very strong (reliable data share of 82% or more) across all evaluated factors. In the case of OCTA2 vs. FA, the level of agreement for the Outline of FAZ factor was assessed as strong, while the agreement for the remaining factors was recorded at a moderate level (reliable data share of 35–63%).

#### 3.2.11. Adverse Events Following Fluorescein Angiography

The independence test (*N* = 98) showed no statistically significant association between sex and the occurrence of adverse events (χ^2^ = 0.364, *p* = 0.13). Although the frequency distribution of adverse events by sex did not differ significantly, it was observed that adverse events occurred more frequently in men (80%) than in women (20%). Overall, adverse events were reported in 5.1% of cases (5 out of 98 study participants).

### 3.3. Implementation of the Remaining Research Objectives

#### 3.3.1. The Impact of Central Retinal Thickness on Best-Corrected Visual Acuity

Pearson’s correlation analysis between BCVA and CRT in the OCT examination revealed a statistically significant negative correlation of weak strength (*r* = −0.37, *p* < 0.001). The negative direction of the association indicates that an increase in CRT was associated with a deterioration in BCVA.

#### 3.3.2. The Impact of Maximum Retinal Thickness on Best-Corrected Visual Acuity

Pearson’s correlation analysis between BCVA and maximum retinal thickness (max RT) in the OCT examination revealed a statistically significant negative correlation of moderate strength (*r* = −0.43, *p* < 0.001). The negative direction of the association indicates that an increase in max RT was associated with a deterioration in BCVA.

#### 3.3.3. The Impact of FAZ Area on Best-Corrected Visual Acuity

Pearson’s *r* correlation was used to assess relationships between variables measured on a ratio scale. For OCTA FAZ 1 versus BCVA, the correlation coefficient (r) = −0.76 (*p* < 0.001); for OCTA FAZ 2 versus BCVA, r = −0.75 (*p* < 0.001); and for FA FAZ versus BCVA, r = −0.76 (*p* < 0.001). A significant negative association of strong magnitude was observed between FAZ area and visual acuity. An increase in FAZ area, as measured by both OCTA and fluorescein angiography (FA), was associated with a deterioration in BCVA.

#### 3.3.4. The Impact of Glycemic Control on the Number of Microaneurysms in the Macula

Pearson’s r correlation was employed to evaluate the relationships among variables measured on a ratio scale. The Pearson correlation coefficients and associated *p*-values for the variable pairs were as follows: OCTA MAs 1 versus HbA1c (r = 0.33, *p* < 0.001), OCTA MAs 2 versus HbA1c (r = 0.60, *p* < 0.001), and FA MAs versus HbA1c (r = 0.54, *p* < 0.001). Based on the collected data, significant positive associations were observed between OCTA MAs 1, OCTA MAs 2, FA MAs, and HbA1c, with the strength of these relationships ranging from weak to moderate. Increases in HbA1c concentration were associated with an increase (from weak to moderate) in the number of MAs detected in OCTA and FA examinations. OCTA MAs 2 exhibited the strongest correlation with HbA1c, while OCTA MAs 1 showed the weakest correlation.

#### 3.3.5. The Impact of Macular Plexus Vessel Density on Best-Corrected Visual Acuity (BCVA)

As the method for correlating variables measured on interval/ratio scales, Pearson’s r correlation was employed. For the variable pair OCTA VD1 versus BCVA, the correlation coefficient (r) was 0.73 (*p* < 0.001), and for OCTA VD2 versus BCVA, r was 0.74 (*p* < 0.001). All examined variable pairs exhibited significant positive correlations ranging from moderate to high. A reduction in VD in both the SCP and DCP resulted in a significant deterioration in BCVA.

#### 3.3.6. The Impact of Macular Plexus Vessel Density on the Evaluation of the “Capillary Loss” Parameter According to the ETDRS Report No. 11

As the method for correlating variables measured on interval/ratio scales, Pearson’s r correlation was used. For OCTA VD1 versus OCTA Capillary Loss 1, a significant negative correlation was found (r = −0.56, *p* < 0.001). Similarly, OCTA VD2 versus OCTA Capillary Loss 1 yielded r = −0.52 (*p* < 0.001). For OCTA VD1 versus OCTA Capillary Loss 2, the correlation was r = −0.54 (*p* < 0.001), and for OCTA VD2 versus OCTA Capillary Loss 2, r = −0.56 (*p* < 0.001). Additionally, OCTA VD1 versus FA Capillary Loss showed r = −0.51 (*p* < 0.001), while OCTA VD2 versus FA Capillary Loss revealed r = −0.54 (*p* < 0.001). The data indicates that all examined pairs of variables exhibited significant moderate negative correlations. A reduction in VD in both the SCP and DCP resulted in a significant moderate increase in the degree of capillary loss, as assessed by both OCTA and FA according to the ETDRS Report No. 11 classification.

#### 3.3.7. The Influence of the Connections Between the Vascular Plexus of the Macula, as Seen in OCTA, on the Size of the FAZ and Best-Corrected Visual Acuity

As the method for correlating variables measured on nominal and ratio scales, the point-biserial correlation (rₚb) was used. For BCVA versus shunts, the point-biserial correlation was −0.33 (*p* < 0.001). For OCTA FAZ 1, OCTA FAZ 2, and FA FAZ versus shunts, the point-biserial correlations were all 0.55 (*p* < 0.001). It was found that the presence of shunts between the SCP and DCP significantly reduced BCVA while also significantly increasing the FAZ area in both FA and OCTA examinations.

#### 3.3.8. Impact of Arterial Hypertension and Tobacco Smoking on the Size of the Foveal Avascular Zone (FAZ)

The mean values of the analyzed variables, stratified by hypertension and smoking status, were as follows:

OCTA FAZ 1: no hypertension, non-smokers (*n* = 14): median = 0.49 (IQR = 0.43); no hypertension, smokers (*n* = 7): median = 0.41 (IQR = 0.16); with hypertension, non-smokers (*n* = 54): median = 0.36 (IQR = 0.37); with hypertension, smokers (*n* = 23): median = 0.41 (IQR = 0.41).

OCTA FAZ 2: no hypertension, non-smokers (*n* = 14): median = 0.50 (IQR = 0.48); no hypertension, smokers (*n* = 7): median = 0.42 (IQR = 0.16); with hypertension, non-smokers (*n* = 54): median = 0.41 (IQR = 0.34), With hypertension, smokers (*n* = 23): median = 0.43 (IQR = 0.43)

AF FAZ: no hypertension, non-smokers (*n* = 14): median = 0.46 (IQR = 0.42); no hypertension, smokers (*n* = 7): median = 0.39 (IQR = 0.15); with hypertension, non-smokers (*n* = 54): median = 0.35 (IQR = 0.39); with hypertension, smokers (*n* = 23): median = 0.38 (IQR = 0.38).

For the FAZ variables, healthy non-smoking subjects exhibited the largest FAZ area. FAZ area decreased in the presence of smoking and/or hypertension. Non-smoking individuals with hypertension had the smallest FAZ area.

For OCTA FAZ1, a Mann–Whitney U test for independent samples showed that the difference between the median OCTA FAZ1 in non-smoking normotensive subjects (Mdn = 0.49; IQR = 0.43) and in smoking hypertensive subjects (Mdn = 0.41; IQR = 0.41) was not statistically significant (U = 154, *p* = 0.839), with a negligible effect size (r = −0.04).

A Mann–Whitney U test likewise demonstrated no significant difference between non-smoking normotensive subjects (Mdn = 0.50; IQR = 0.48) and smoking hypertensive subjects (Mdn = 0.43; IQR = 0.43) for OCTA FAZ2 (U = 155.5, *p* = 0.876; r = −0.04).

Finally, the Mann–Whitney U test indicated no significant difference in AF FAZ between non-smoking normotensive subjects (Mdn = 0.46; IQR = 0.42) and smoking hypertensive subjects (Mdn = 0.38; IQR = 0.38) (U = 153.5, *p* = 0.826; r = −0.05).

## 4. Discussion

DME is an escalating public health concern. The projected rise in diabetes prevalence over the coming years places considerable pressure on investigators to identify effective disease control strategies, enable early detection of diabetic complications, and optimize therapeutic approaches [16]. DME represents the most frequent cause of visual acuity decline in patients with diabetic retinopathy [17], and its principal ophthalmic risk factor is the severity of underlying retinopathy [18]. From an economic standpoint, since many affected individuals are of working age, DME can lead to temporary work absence or even permanent withdrawal from professional activity.

Identification of DME risk factors has spurred randomized clinical trials linking systemic disease control to retinopathy progression. The Diabetes Control and Complications Trial (DCCT) demonstrated that intensive glycemic control in type 1 diabetes reduced the 9-year cumulative incidence of DME by 29% [19]. Likewise, the United Kingdom Prospective Diabetes Study (UKPDS) in type 2 diabetes showed that stringent blood glucose management decreased the need for retinal laser treatment attributable to DME [20]. Tang et al. analyzed 434 OCTA scans of the superficial capillary plexus from patients across various retinopathy stages to evaluate potential biomarkers. They found that OCTA parameters correlated with retinopathy severity but not with the presence of DME and that systemic factors such as blood pressure, lipid levels, and Body Mass Index did not significantly correlate with OCTA metrics [21]. In our study, neither hypertension nor smoking history exerted a statistically significant effect on FAZ dimensions as measured by FA or OCTA.

Nonproliferative diabetic retinopathy partly relies on both the presence and distribution of microaneurysms (MAs) on fundus examination [22]. Although many MAs can be detected by OCTA, in our study, the combined mean MAs count in the SCP and DCP (3.24) remains lower than that seen with FA (5.47; *p* < 0.001). Nonetheless, a very strong correlation exists between MA counts in OCTA and FA, particularly between DCP-derived OCTA counts and FA results, even though perfect one-to-one MA concordance is lacking. This suggests that certain lesions seen on OCTA do not correspond precisely to those on FA and vice versa. Consistent with other reports, Ishibazawa et al. localized most MAs to the deep capillary plexus on OCTA, implying a DCP origin [23,24,25]. Histological and OCT studies have shown that MAs predominantly reside within the inner nuclear layer and at its inner and outer boundaries [26,27]. Ishibazawa and colleagues also highlighted that hyperfluorescent spots on FA do not always represent true MAs but may instead reflect focal leakage from pathological capillaries, whereas punctuation signals exclusive to OCTA could correspond to capillary termini or vertically oriented capillaries [23]. Moreover, smaller 3 × 3 mm OCTA scans may underestimate MA detection compared to larger 6 × 6 mm scans [28], and slow or absent erythrocyte flow within some MAs may fall below the SSADA flow detection threshold of 0.3 mm/s [29]. Histologic evidence further confirms that nonperfused MAs can contain multinucleated cells rather than erythrocytes [30]. Clinically, most MAs on FA are conspicuous on first inspection, whereas OCTA-identified MAs often require more deliberate scrutiny—a finding corroborated by other investigators [31]. The combination of rapid interpretation and higher sensitivity of FA in MAs detection makes it potentially a more suitable tool for the accurate staging of nonproliferative diabetic retinopathy and for assessing treatment response.

A key objective of our study was to compare FAZ area measurements in DME patients by OCTA versus FA. Early work by Bresnick et al. first documented FAZ enlargement in diabetic retinopathy using FA [32], and Mansour et al. demonstrated progressive FAZ expansion with advancing retinopathy, culminating in ischemic maculopathy [33]. In the present series, FAZ area differed significantly between OCTA and FA, yet the two modalities exhibited high intermethod correlation, especially between the OCTA deep plexus FAZ and the FA-derived FAZ. Furthermore, FAZ measurements were consistently larger on OCTA. Within OCTA itself, FAZ area differed significantly between the superficial and deep plexuses, with the DCP showing greater values—a result aligned with other studies [34,35]. Our cohort’s mean FAZ dimensions differed from those in prior reports, likely reflecting distinct inclusion criteria [35,36]. Beyond quantitative metrics, subjective evaluation of FAZ border definition favored OCTA, as FA images can suffer from vascular masking by dye leakage and background fluorescence. Macular edema could potentially impair the precise demarcation of FAZ on OCTA and FA. Intraretinal fluid accumulation and distortion of foveal contour can potentially mislead both computer algorithms and researchers, resulting in apparent enlargement or irregularity of the FAZ [37,38]. A 3 × 3 mm OCTA scan demonstrated practical utility in detecting macular ischemic changes, echoing findings from others [34]. FAZ measurement remains invaluable in clinical practice for diagnosing and monitoring ischemic maculopathy. Given OCTA’s broader availability, rapid acquisition, and lack of adverse reactions, it represents a valuable noninvasive tool for identifying macular ischemia in DME patients.

Despite FA’s status as the clinical gold standard for ischemic maculopathy imaging, the standardized ETDRS report 11 classifications enabled us to assess whether OCTA could serve as a noninvasive FA alternative in DME with ischemic components. Intermethod agreement ranged from moderate to very strong, and it was greatest when comparing FA with OCTA SCP results, as also noted by Bradley et al. [39], although Cennamo et al. reported stronger DCP correlations [40]. Subjectively, ischemic zones appeared far clearer on OCTA, unencumbered by dye-leakage masking. FA cannot reliably depict the DCP due to scattered light from deeper retinal layers obscuring capillary detail [41]. In contrast, OCTA visualizes vessel caliber, morphology, and flow changes in both superficial and deep plexuses corresponding to ischemic maculopathy [42]. Overall, given the high ETDRS classification concordance—particularly between FA and OCTA SCP—OCTA emerges as a clinically useful modality for diagnosing ischemic components in DME. With further prospective standardization, OCTA may ultimately reduce reliance on conventional FA.

We also quantified vascular density (VD) in both OCTA plexuses and found a strong, statistically significant correlation between decreasing VD and worsening best-corrected visual acuity (BCVA). Rodrigues et al. observed a similar correlation, albeit only in the SCP and with a lower magnitude (r = 0.375 vs. r = 0.73) [43], possibly reflecting our more homogeneous sample. VD was lower in the SCP than in the DCP, consistent with other diabetic cohorts [23].

Glycated hemoglobin (HbA1c) percentage, reflecting average glycemia over the preceding 2–3 months [44], is recommended for diabetes diagnosis and monitoring, particularly in type 2 diabetes [45]. The ACCORD trial demonstrated that intensive glycemic control in type 2 diabetics (mean HbA1c 6.4% vs. 7.5%) reduced retinopathy progression by 35% over four years (≥3-step ETDRS change) [46]. Studies conflict regarding HbA1c’s effect on FAZ metrics: Gozlan et al. and others reported favorable FAZ parameter associations with lower HbA1c in nonproliferative retinopathy [47,48], whereas Conrath et al. and other authors found no such correlation [49,50]. In our cohort, FAZ area did not differ significantly between patients with poor glycemic control (HbA1c > 7.0%) and those with good control (≤7.0%). However, MA counts—both by OCTA plexus and FA total—correlated positively with higher HbA1c; the strongest association was in the OCTA DCP.

Retinal capillary network function and morphology underpin neuronal metabolism, requiring efficient oxygen delivery and metabolite clearance. Histology identifies three distinct retinal capillary plexuses—superficial (SCP), intermediate (MCP), and deep (DCP) [51]—plus a fourth radial peripapillary plexus in the nerve-fiber layer [52]. Vertical anastomoses connect these networks and are visible on OCTA [53,54]; in our study, shunt visualization was inconsistent, but their presence correlated significantly with worse BCVA and larger FAZ areas by both OCTA and FA. Retinal hypoxia in advanced diabetic retinopathy may induce angiogenesis and upregulate vascular endothelial growth factor (VEGF), promoting endothelial proliferation [55], which may explain the higher shunt prevalence in advanced disease. OCTA vessel density could be a useful biomarker for visual acuity prognosis and treatment response.

Regarding safety, OCTA is noninvasive and well tolerated [56], whereas FA carries invasive procedural risks from intravenous fluorescein, most commonly mild nausea [57,58]. In our study, adverse events occurred in 5.1% as transient nausea and resolved without intervention. This incidence seems to be comparable with results from other studies: 2.9% [58] and 6.83% [57]. OCTA is a safer choice, in particular for patients with multiple comorbidities and allergy to fluorescein.

Beyond the limitations already discussed, additional constraints should be acknowledged. First, accurate MA identification on OCTA versus FA remains challenging without histopathologic confirmation. Although MA density is prognostic for clinically significant DME progression [59,60], OCTA’s noninvasive, repeatable imaging allows longitudinal DME and retinopathy monitoring despite underdetection of some MAs. Second, flow artifacts could have influenced our results. In OCTA, false negative flow artifacts [61], often called shadowing artifacts, could be caused by cystic spaces, hemorrhages, exudates, or cotton wool spots—common in DR. These artifacts can affect FAZ assessment, making it more difficult to evaluate its size and regularity, and can lead to underestimation of total MA count and vessel density. DCP scans are particularly susceptible to those errors. Compared to SCP, the signal has to go through more layers where it may be scattered, absorbed, or attenuated—especially in the presence of retinal thickening—which further hinders signal return from macula to OCTA detector. Additionally, the DCP is more prone to software-based detection errors due to its smaller, tightly packed capillaries, which are more easily missed by OCTA algorithms.

Although less common in cases of DR, projection artifacts from the superficial plexus may create false-positive flow signals in the DCP, leading to an overestimation of vessel density [61]. Third, both negative and positive flow artifacts, as well as poor scan quality, can cause segmentation errors, potentially resulting in either under- or overestimation of perfusion. OCTA software often enables manual correction of this error. We reduced artifact risk by ensuring high-quality scans (SSI > 37). FA-specific artifacts, which could influence the results, include leakage or pooling in cystic spaces, obscuring details and making it harder to asses the FAZ border, visualize capillary dropout, or identify microaneurysms [62].

Fourth, the small 3 × 3 mm en face OCTA scan, while adequate for foveal SCP and DCP assessment, limited evaluation of peripheral nonperfusion reaching the scan margins. This is an important disadvantage as peripheral retinal ischemia is a well-recognized marker of diabetic retinopathy severity and progression. These issues may be addressed by employing wide-field OCTA scans, which can image a retinal area of 12 mm × 12 mm—four times larger than the standard 3 × 3 mm or 6 mm × 6 mm scans [63]. Recent studies have described widefield FA and OCTA systems covering 15 × 15 mm [64] or even 24 × 20 mm^2^ [65] of retina, enabling assessment of peripheral vascular pathology. Widefield acquisitions typically require longer examination time, a montage of OCTA scans, and may be more susceptible to motion and segmentation artifacts. In contrast, a 3 × 3 mm scan offers advantages in the DME assessment, including faster acquisition time, reduced motion artifacts, improved image quality, and higher spatial resolution. These features still make it a good tool for screening and quantifying vessel density and foveal microvascular changes. In our study, motion artifacts excluded 5.7% of OCTA scans. In comparison, Ho et al. observed artifacts in 13% of 3 × 3 mm scans, with no significant artifact rate difference between 3 × 3 mm and 6 × 6 mm protocols [28]. Nevertheless, we believe that with ongoing advancements in acquisition speed, resolution, and single-capture technology, widefield OCTA is likely to become a crucial modality for comprehensive DR evaluation in the future.

In summary, this study provides evidence that OCTA may serve as a viable alternative to conventional FA in certain clinical indications. Nonetheless, both imaging methods are complementary for a comprehensive assessment of diabetic retinopathy activity and DME.

## 5. Conclusions

The OCTA and FA results demonstrate a high degree of correlation in both delineating the dimensions of FAZ and quantifying microaneurysms. OCTA is less sensitive for the detection of macular MAs. Poor glycemic control is correlated with an increased number of microaneurysms in the macula. FAZ measurements appears larger when evaluated by OCTA than by FA, with OCTA providing sharper delineation of its borders. An enlarged FAZ area and a reduction in the VD of both SCP and DCP are associated with diminished VD. OCTA may be valuable in evaluating selected parameters of macular ischemia, and wider scans would allow for more comprehensive diabetic retinopathy control. The disadvantages of FA in diagnosing DME include its prolonged duration, invasiveness, and the potential risk of adverse reactions following contrast administration. OCTA represents a straightforward, rapid, noninvasive, and reproducible imaging modality. Both methods are susceptible to artifacts that hinder result interpretation, especially in the presence of DME.

## Figures and Tables

**Table 1:** Presents the descriptive statistics of the study participants along with the results of the Shapiro–Wilk test (*N* = 98).

	*N*	*M*	*SD*	*Mdn*	*Min*	*Max*	*Sk.*	*Kurt.*	*W*	*p*
Age	98	63.97	7.11	65	36	76	−1.22	2.68	0.92	<0.001
BCVA	98	0.55	0.14	0.5	0.2	0.8	−0.28	0.17	0.9	<0.001
CRT	98	427.3	109.25	404	220	790	1.3	2.25	0.9	<0.001
Max RT	98	487.24	102.11	465.5	332	790	1.05	0.9	0.91	<0.001
HbA1c	98	7.8	1.48	7.55	5.2	11.4	0.53	0.01	0.96	0.009
OCTA FAZ 1	98	0.49	0.33	0.41	0.09	1.98	2.03	5.95	0.82	<0.001
OCTA FAZ 2	98	0.52	0.33	0.43	0.14	2.01	2.12	6.4	0.81	<0.001
OCTA VD 1	98	46.43	5.87	46.45	31.9	57.9	−0.24	−0.23	0.98	0.231
OCTA VD 2	98	47.27	5.74	47.3	32.4	59.6	−0.2	−0.04	0.98	0.297
OCTA MAs 1	98	0.84	1.6	0	0	14	5.91	46.95	0.46	<0.001
OCTA MAs 2	98	2.41	2.27	2	0	8	0.55	−0.85	0.88	<0.001
AF FAZ	98	0.46	0.32	0.38	0.09	1.99	2.11	6.44	0.81	<0.001
AF MAs	98	5.47	4.93	5	0	17	0.48	−0.96	0.9	<0.001
Diabetes	98	15.21	8.7	15	0.7	35	0.11	−0.62	0.96	0.015
Vision	98	10.08	12.44	6	1	60	2.5	6.2	0.66	<0.001

Age (in years); BCVA—best corrected visual acuity; CRT—central retinal thickness (in µm); Max RT—maximum retinal thickness (in µm); HbA1c—concentration of glycosylated hemoglobin; OCTA FAZ 1—foveal avascular zone measured in OCTA superficial plexus scans; OCTA FAZ 2—foveal avascular zone measured in OCTA deep plexus scans; OCTA VD 1—vessel density measured in OCTA superficial plexus scans; OCTA VD 2—vessel density measured in OCTA deep plexus scans; OCTA MAs 1—microaneurysms number measured in OCTA superficial plexus scans; OCTA MAs 2—microaneurysms number measured in OCTA deep plexus scans; FA FAZ—foveal avascular zone measured in FA superficial plexus scans; FA MAs—microaneurysm number measured in OCTA deep plexus scans; Diabetes—duration of diabetes (in years); Vision—duration of vision impairment (in months); *N*—number of patients; M—mean; SD—standard deviation; Min—minimum value; Max—maximum value; Sk—skewness; Kurt—kurtosis; W—Shapiro–Wilk statistic; *p*—significance level of the statistic.

## Data Availability

The original contributions presented in this study are included in the article. Further inquiries can be directed to the corresponding author.

## Abbreviations

Anty-VEGF	vascular endothelial growth factor inhibitor
BCVA	best corrected visual acuity
CRT	central foveal thickness
DCP	deep capillary plexus
DD	disc diameter
DME	diabetic macular edema
DR	diabetic retinopathy
DRIL	disorganization of retinal inner layers
EDTA	ethylenediaminetetraacetic acid
ETDRS	Early Treatment Diabetic Retinopathy Study
FA	fluorescein angiography
FAZ	foveal avascular zone
HbA1c	glycated hemoglobin
HPLC	high-performance liquid chromatography
ILM	internal limiting membrane
IRMA	intraretinal microvascular abnormalities
MAs	microaneurysms
Max RT	maximum retinal thickness
MCP	middle capillary plexus
NGSP	National Glycohemoglobin Standardization Program
NVD	neovascularization at the disc
NVE	neovascularization elsewhere
OCT	optical coherence tomography
OCTA	optical coherence tomography angiography
OGTT	oral glucose tolerance test
SCP	superficial capillary plexus
SD-OCT	spectral domain optical coherence tomography
SRF	subretinal fluid
SSADA	split spectrum amplitude decorrelation angiography
SSI	signal strength index
VD	vessel density
VEGF	vascular endothelial growth factor
